# Loss of TGR5-activating bile acids is associated with disease activity in inflammatory bowel disease

**DOI:** 10.1038/s41598-026-63156-0

**Published:** 2026-07-21

**Authors:** Johannes Stallhofer, Julia Leonhardt, Juliane Semmler, Sophie Neugebauer, Michael Kiehntopf, Wiebke Löhden, Lea Homeister, Martin Ungelenk, Christian A. Hübner, Arndt Steube, Silvio Waschina, Andreas Stallmach

**Affiliations:** 1 Department of Internal Medicine IV, Jena University Hospital, Am Klinikum 1, D-07747 Jena, Germany; 2 Department of Anesthesiology and Intensive Care Medicine, Jena University Hospital, D-07747 Jena, Germany; 3 Institute of Clinical Chemistry and Laboratory Diagnostics, Centralized Diagnostic Laboratory Services, Jena University Hospital, D-07747 Jena, Germany; 4 Institute of Human Genetics, Jena University Hospital, D-07747 Jena, Germany; 5 Institute of Human Nutrition and Food Science, Kiel University, Heinrich-Hecht-Platz 10, D-24118 Kiel, Germany

**Keywords:** Bile acids, TGR5 bioactivity, Microbiota, Inflammatory bowel disease, Crohn´s disease, Ulcerative colitis, Gastroenterology, Microbiology

## Abstract

The gut microbiota communicates extensively with its host through small metabolites, such as bile acids. Primary bile acids are synthesized by the host and secreted into the intestine, where they are actively converted by the microbiota into secondary bile acids. Depending on the resulting bile acid composition, the host’s bile acid receptor, Takeda G protein–coupled receptor 5 (TGR5), is activated and mediates immune tolerance. It has been suggested that a disturbed bile acid profile in inflammatory bowel disease (IBD) might lead to inflammation via reduced activation of TGR5. Our study is the first to investigate whether bile acid-induced TGR5 activation differs between healthy individuals and patients with IBD. Bile acid profiles in stool and plasma were quantified by mass spectrometry, and TGR5 bioactivity was assessed from these profiles. In parallel, metagenomic sequencing was performed on fecal samples. We demonstrate that reduced alpha diversity in IBD is associated with a loss of microbial capacity for bile acid transformation, resulting in a significantly decreased secondary-to-primary bile acid ratio (sBA/pBA) in both stool and circulation. TGR5 bioactivity induced by bile acid profiles was substantially reduced in IBD patients, and a lower TGR5 bioactivity correlated with increased inflammatory activity.

**Supplementary Information:**

The online version contains supplementary material available at 10.1038/s41598-026-63156-0.

## Introduction

Inflammatory bowel disease (IBD), encompassing Crohn’s disease (CD) and ulcerative colitis (UC), is thought to result from a complex interplay of genetic predisposition, shifts in the gut microbiota, and a dysregulated host immunity^1,2^. However, the exact pathogenesis remains unknown. Recent metagenomic and metatranscriptomic analyses have provided detailed insights into the altered gut microbiome in IBD^3^. Common features of IBD-associated dysbiosis include a reduction in overall taxonomic diversity and a taxonomic shift toward a reduction in obligate anaerobic bacteria, such as *Faecalibacterium prausnitzii*, and increased abundances of facultative anaerobes. An altered gut microbiome composition, in conjunction with a concomitant alteration in the community metabolism, contributes to the risk of microbiome-induced pro-inflammatory responses in the preclinical phase of IBD^4^. In particular, bile acid metabolism has gained attention for its role in mediating host-microbiome interactions and inflammatory responses^5^. Bile acids, chemically modified by gut microbial enzymes, influence intestinal immunity and inflammation by activating host receptors such as farnesoid X receptor (FXR) and Takeda G protein–coupled receptor 5 (TGR5), and are increasingly recognized as a mechanistic link between microbial dysbiosis and IBD pathogenesis^6^. In line with this, many authors proposed that the TGR5 activity induced by human bile acid profiles might be reduced in IBD^7–10^.

After hepatic synthesis, primary bile acids such as cholic acid (CA) and chenodeoxycholic acid (CDCA) are secreted into the small intestine, where they facilitate lipid emulsification and absorption. A fraction of these primary bile acids reaches the colon, where they are biotransformed by specific gut bacteria. A pivotal microbial conversion is 7α-dehydroxylation, a metabolic pathway that removes the 7α-hydroxyl (OH) group from CA and CDCA, converting them into the secondary bile acids deoxycholic acid (DCA) and lithocholic acid (LCA), respectively^11^. This reaction is predominantly catalyzed by anaerobic bacteria of the genus *Clostridium*—notably *Clostridium scindens*, *Clostridium hiranonis*, and *Clostridium hylemonae*—which express the bile acid-inducible (bai) operon, a gene cluster encoding the enzymatic machinery required for this transformation. It has been suggested that only about 0.0001% of cultivable colonic bacteria can catalyze this reaction, primarily members of the *Clostridium*genus^12^.

However, recent metagenomic analyses have identified the previously considered “uncultivable” Firmicutes CAG-103 as the predominant bai gene–carrying taxon in healthy individuals, whereas cultivable bile acid 7α-dehydroxylating species, such as *Clostridium scindens*, appear to be enriched in the context of IBD-associated inflammation^13^. Changes in the gut microbiome correspond with disturbed microbial metabolic functions in IBD patients^14^, influencing both the composition of the bile acid pool and its systemic effects on the host. In the immune system, bile acids are recognized for their ability to inhibit the production of inflammatory cytokines^15,16^. Microbiota-modified bile acids interact with host receptors, most notably TGR5, also known as GPBAR1 (G protein–coupled bile acid receptor 1). TGR5 is a membrane-bound G protein–coupled receptor endogenously activated by bile acids. Since its discovery, TGR5 has been increasingly recognized as a critical regulator in various physiological and pathophysiological processes, particularly those related to energy and glucose metabolism, bile acid homeostasis, and inflammatory and neoplastic diseases^17^.

In macrophages and other immune cells, TGR5 inhibits NF-κB-dependent transcription of pro-inflammatory cytokines, thereby exerting anti-inflammatory effects. Studies using gene-ablated mice have shown that FXR and TGR5 are essential for maintaining a tolerogenic immune phenotype in the intestine, and their deletion leads to the polarization of intestinal T cells and macrophages toward a pro-inflammatory profile^18^. This mechanism is particularly relevant in the pathophysiology of IBD.

Against this background, we conducted metagenomic sequencing of fecal samples from patients with Crohn’s Disease and ulcerative colitis, and healthy controls. Bile acids were quantified by mass spectrometry in both stool and plasma samples obtained from the same individuals. Alterations in the bile acid profile were analyzed with respect to their potential immunosuppressive activity via TGR5 signaling. We demonstrated that a decreased ratio of secondary-to-primary bile acids (sBA/pBA) in IBD patients was associated with a reduced abundance of secondary bile acid (sBA)-producing bacteria. This deficiency in microbial metabolic pathways responsible for sBA production likely contributes to intestinal inflammation in IBD by reducing the immunosuppressive TGR5 bioactivity.

## Results

### Stool and plasma bile acid levels

**Fig. 1:**
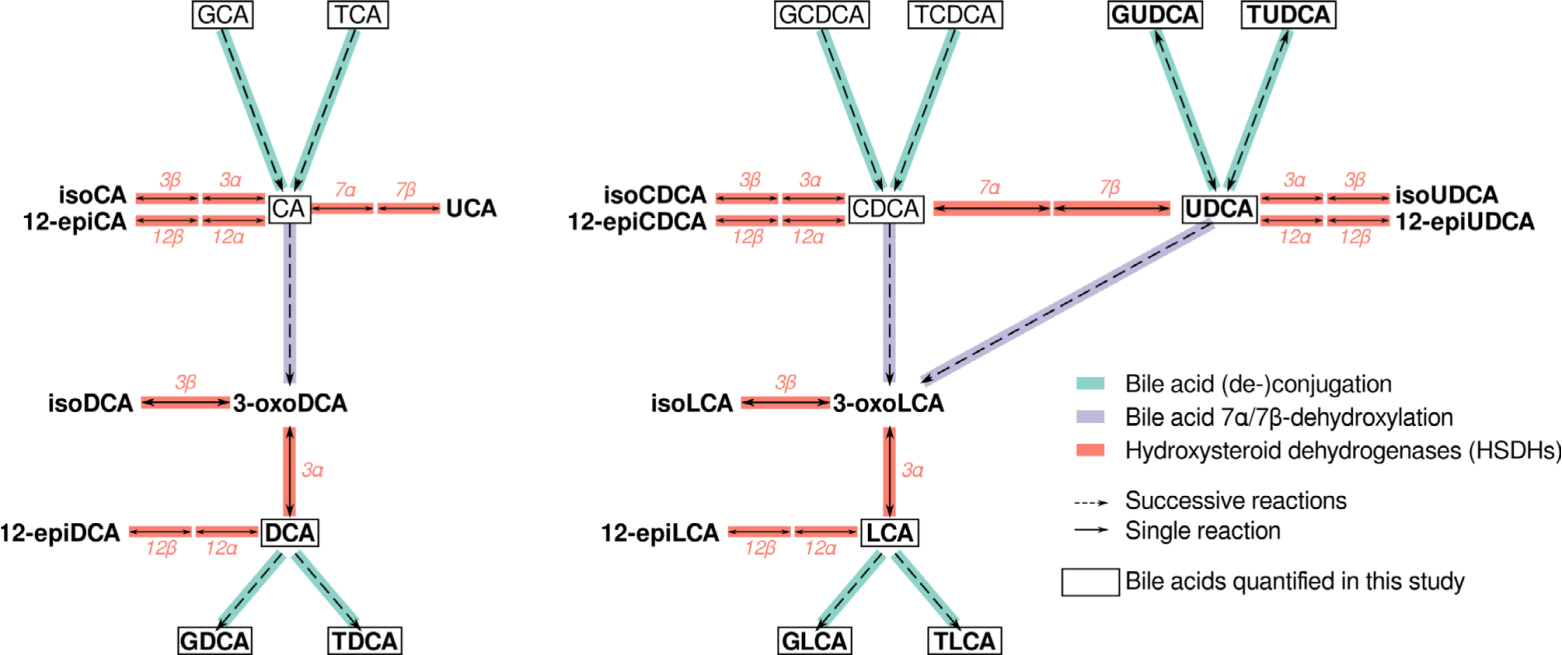
Gut microbial pathways for bile acid transformations. Framed bile acids were quantified in stool and plasma samples in this study. Dashed arrows indicate pathways that include two or more reactions and enzymes. Single reactions are displayed as solid arrows. Hydroxysteroid dehydrogenase reactions are highlighted in red and labeled with the C-position in the bile acid structure (i.e., 3, 7, or 12) and the transformation type (alpha or beta). Abbreviations of bile acids are explained in Table 2.

**Fig. 2:**
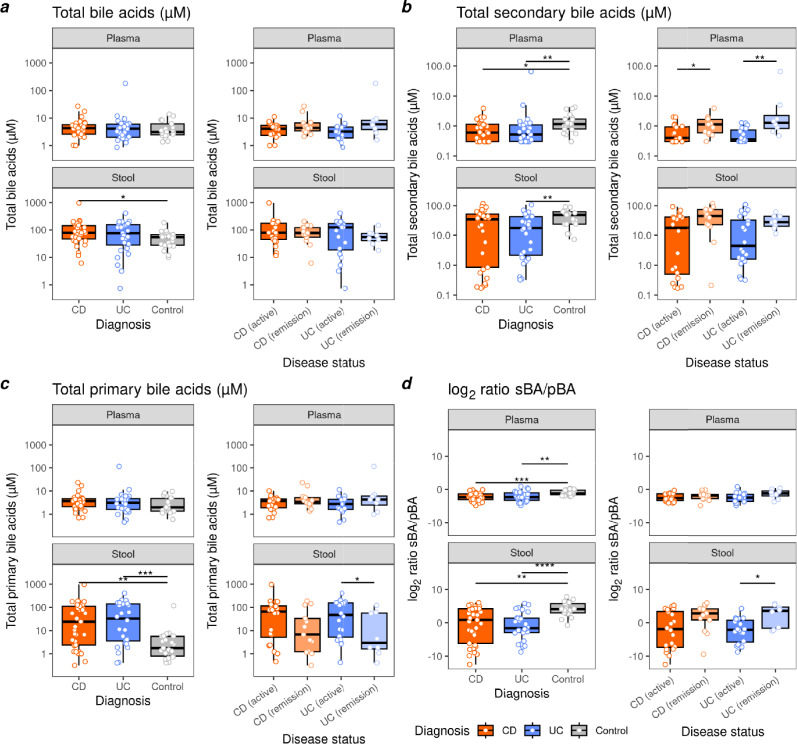
Aggregated bile acid metrics in plasma and stool samples. (**a**) Concentration of total bile acids, (**b**) concentration of total secondary bile acids (sBAs), (**c**) concentration of total primary bile acids (pBAs), (**d**) log_2_-ratio of sBAs to pBAs. Bars above box plots indicate significant differences between the two groups at each end of the bar (Wilcoxon rank-sum test with continuity correction). In each panel, the data are stratified by diagnosis (Crohn’s Disease [CD], ulcerative colitis [UC], and healthy controls [Control]; left plots) and stratified by disease status (active disease vs remission; right plots). Significance levels: * (p < 0.05), ** (p < 0.01), *** (p < 0.001), **** (p < 0.0001).

Patients with a confirmed diagnosis of Crohn’s disease (CD, n=33) or ulcerative colitis (UC, n=28) in either active disease flare or remission were included in the study and compared to 21 healthy controls (Table 1). Fifteen individual bile acids were quantified in stool and plasma samples (Fig. 1 and Table 2). In plasma, total bile acid concentrations did not differ significantly between healthy controls and patients with CD or UC, nor between patients with active disease and those in remission (Wilcoxon rank-sum test, *p*_*CD*_ = 0.4759, *p*_*UC*_ = 0.9344, Fig. 2a). In contrast, the total plasma concentration of secondary bile acids (sBA) was significantly reduced in both CD and UC compared to healthy controls (*p*_*CD*_ = 0.0101, *p*_*UC*_ = 0.0053, Fig. 2b). Moreover, within each disease group, patients with active CD or UC exhibited significantly lower plasma secondary bile acid levels than those in remission (*p*_*CD*_ = 0.0244, *p*_*UC*_ = 0.0021, Fig. 2b). This reduction in sBA was also evident when analyzing individual secondary bile acids: plasma levels of deoxycholic acid (DCA, FDR-adjusted *p*_*CD*_ = 0.0031, adj. *p*_*UC*_ = 0.0009), its conjugated forms glycodeoxycholic acid (GDCA, adj. *p*_*CD*_ = 0.0031, adj. *p*_*UC*_ = 0.0022) and taurodeoxycholic acid (TDCA, adj. *p*_*CD*_ = 0.0031, adj. *p*_*UC*_ = 0.0057), as well as lithocholic acid (LCA, adj. *p*_*CD*_ = 0.0062, adj. *p*_*UC*_ = 0.01) and its glycine-conjugated form glycolithocholic acid (GLCA, adj. *p*_*CD*_ = 0.0031, adj. *p*_*UC*_ = 0.0057), were significantly decreased in both CD and UC compared to controls (Fig. S1a). In ulcerative colitis specifically, plasma levels of GDCA and glycoursodeoxycholic acid (GUDCA) were significantly lower in those with active disease compared to those in remission (GDCA: *p*_*UC*_ = 0.0383, GUDCA: *p*_*UC*_ = 0.0395, Fig. S1b). No significant differences were observed in plasma total primary bile acids (pBA) between controls and patients with CD or UC, or between disease activity states (Fig. 2c). The ratio of plasma sBA to pBA was significantly lower in both CD and UC patients relative to healthy controls (*p*_*CD*_ = 0.0002, *p*_*UC*_ = 0.0037, Fig. 2d).

**Table 1:** Clinical characteristics of the study cohort. Abbreviations: ADA, adalimumab; BMI, body mass index; GIT, gastrointestinal tract; GOL, golimumab; IFX, infliximab; JAK, janus kinase; 6-MP, 6-mercaptopurine; PSC primary sclerosing cholangitis, TNF, tumor necrosis factor.

	Crohn’s disease (CD)	Ulcerative colitis (UC)	Healthy controls
Total (n)	33	28	21
Active disease (n)	20	20	
Remission (n)	13	8	
Age [years/median]	49.0 (40.0–59.0)	36.5 (31.8–49.8)	30.0 (25.0–36.0)
Age was not comparable across the three groups (one-way ANOVA, p = 0.006).			
Female (n)	20 (60.6%)	13 (46.4%)	11 (52.4%)
The proportion of female patients did not differ significantly among the three groups (60.6%, 71.4%, and 52.4%, respectively; χ^2^ = 1.91, p = 0.38).			
BMI	24.9 (21.0–29.9)	27.3 (23.0–29.5)	22.7 (20.8–24.0)
Mean values did not differ significantly between patients with Crohn’s disease, ulcerative colitis, and controls (one-way ANOVA: F(2,79)=2.87, p=0.063).			
Smokers (n)	6 (18.2%)	3 (10.7%)	0 (0%)
The proportion of current smokers did not differ significantly among the three groups (p = 0.11).			
Antibiotic treatment during the last 3 months	6	7	2
Montreal classification	L1 (terminal ileum): n=3	E1 (proctitis): n=0	
	L2 (colon): n=12	E2 (left-sided colitis): n=8	
	L3 (ileocolon): n=15	E3 (pancolitis): n=20	
	L4 (upper GIT): n=1		
	L4+ (upper GIT+distal disease): n=2		
Resections	Hemicolectomy: n=3	Hemicolectomy: n=1	
	Ileocecal resection: n=6	Ileocecal resection: n=1	
PSC	**n=0**	**n=1**	
Medication	n=2	n=18	
mesalazine	n=1	n=6	
budesonide	n=3	n=7	
steroids	n=5	n=5	
azathioprine/6-MP	n=1	n=0	
methotrexate	n=9	n=6	
anti-TNF (IFX, ADA, GOL)	n=1	n=4	
vedolizumab	n=15	n=5	
ustekinumab JAK inhibitors (tofacitinib)	n=0	n=1

**Table 2:** Circulating bile acids quantified in this study. Values for TGR5 activation potency are taken from Leonhardt et al.^19^. Values for bile acids are given in µmol/L.

	Bile acids	TGR5 potency (*P*_*b*_)	Crohn’s disease		Ulcerative colitis			
			Active disease	In remission	Overall	Active disease	In remission	Overall
Primary bile acids	CA	1.0						
	CDCA	8.4	15 (26–29)					
	GCA	1.2						
	GCDCA	7.0						
	TCA	1.4						
	TCDCA	8.1						
Secondary bile acids	DCA	28.0						
	GDCA	18.1						
	GLCA	180.6						
	GUDCA	1.2						
	LCA	28.0						
	TDCA	23.3						
	TLCA	243.5						
	TUDCA	1.8						
	UDCA	1.9						

CA, cholic acid; CDCA, chenodeoxycholic acid; DCA, deoxycholic acid; GCA, glycocholic acid; GCDCA, glycochenodeoxycholic acid; GDCA, glycodeoxycholic acid; GLCA, glycolithocholic acid; GUDCA, glycoursodeoxycholic acid; LCA, lithocholic acid; TCA, taurocholic acid; TDCA, taurodeoxycholic acid; TCDCA, taurochenodeoxycholic acid; TLCA, taurolithocholic acid; TUDCA, tauroursodeoxycholic acid; UDCA, ursodeoxycholic acid.

In contrast to plasma, where alterations were more pronounced in sBA, stool samples showed the most prominent differences in pBA between patients with inflammatory bowel disease (IBD) and healthy controls. Both UC and CD patients exhibited significantly elevated levels of stool total pBA compared to healthy controls (*p*_*CD*_ = 0.0011, *p*_*UC*_ = 0.0004, Fig. 2c), with UC patients in active disease displaying higher stool pBA levels than those in remission (*p*_*UC*_ = 0.0428). This increase was consistent across all quantified pBAs: cholic acid (CA, adj. *p*_*CD*_ = 0.0056, adj. *p*_*UC*_ = 0.0076), chenodeoxycholic acid (CDCA, adj. *p*_*CD*_ = 0.0056, adj. *p*_*UC*_ = 0.0132), glycocholic acid (GCA, adj. *p*_*CD*_ = 0.0024, adj. *p*_*UC*_ = 0.0007), glycochenodeoxycholic acid (GCDCA, adj. *p*_*CD*_ = 0.0024, adj. *p*_*UC*_ = 0.0025), taurocholic acid (TCA, adj. *p*_*CD*_ = 0.0024, adj. *p*_*UC*_ = 0.0001), and taurochenodeoxycholic acid (TCDCA, adj. *p*_*CD*_ = 0.0056, adj. *p*_*UC*_ = 0.0132)—in both UC and CD patients (Fig. S2a). Regarding stool secondary bile acids, levels of deoxycholic acid (DCA) were significantly lower in UC patients compared to controls (adj. *p*_*UC*_ = 0.0154), and lithocholic acid (LCA) was significantly reduced in both CD and UC groups (adj. *p*_*CD*_ = 0.0234, adj. p_*UC*_ = 0.0025, Fig. S2a). The reduction in fecal secondary bile acids tended to be even more pronounced in patients with active disease (Fig. S2b). Similar to plasma, the stool sBA to pBA ratio was significantly decreased in IBD patients relative to healthy controls (*p*_*CD*_ = 0.0013, *p*_*UC*_ = 8.9x10^−6^), with this reduction being particularly marked in individuals with active disease compared to remission (*p*_*CD*_ = 0.0677, *p*_*UC*_ = 0.0114, Fig. 2d).

### Functional impact of bile acid dysmetabolism

**Fig. 3:**
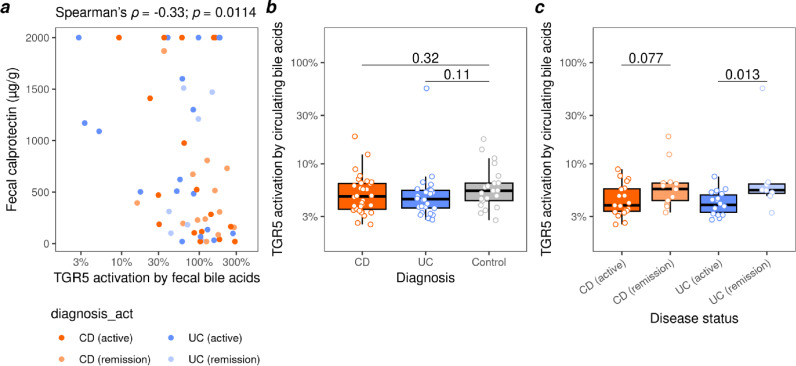
Estimated TGR5 activation by the profile of measured bile acid concentrations. (**a**) Anti-inflammatory effect of Takeda G protein–coupled receptor 5 (TGR5) activation by bile acids measured in stool samples. Estimated TGR5 activation by fecal bile acids correlates negatively with fecal calprotectin levels (Spearman’s rank correlation; rho ($\rho$) = −0.33; *p* = 0.0114). (**b**) Estimated TGR5 activation by circulating (plasma) bile acids stratified by diagnosis (Crohn’s Disease [CD], ulcerative colitis [UC], and healthy controls [Control]). (**c**) Estimated TGR5 activation by circulating bile acids stratified by disease status (active disease vs remission). Bars above box plots indicate significant differences between the two groups at each end of the bar (Wilcoxon rank-sum test with continuity correction) and numeric labels are *p*-values.

To explore the potential consequences of a disturbed bile acid metabolism on the inflammatory response, we analyzed the immunosuppressive effect of both stool and circulating bile acids. Therefore, the activation of the anti-inflammatory receptor TGR5 by bile acids was determined as described before^19,20^ and correlated with fecal calprotectin. In our patients, decreased TGR5 activity by fecal bile acids clearly correlated with increased levels of fecal calprotectin (Spearman’s rank correlation, $\rho$ = −0.33, *p* = 0.0114, Fig 3a).

Likewise, TGR5 activation by plasma bile acids was assessed. A cut-off of 28.3% TGR5 activation has been used previously to distinguish physiological from clearly immunosuppressive bile acid profiles (e.g., as observed in liver failure)^19,20^. Except for one patient with known primary sclerosing cholangitis due to UC (TGR5 activity by plasma bile acids = 55.4%), all patients showed TGR5 activation by circulating bile acids of <28.3% (Fig. 3b), indicating adequate hepatobiliary excretion. Remarkably, patients with active UC showed significantly lower levels of TGR5 activation when compared to UC in remission (Wilcoxon rank-sum test, *p*_*UC*_ = 0.0094, Fig. 3c). A similar effect was also observed for patients with CD (*p*_*CD*_ = 0.0094, Fig. 3c). These results indicate a reduced TGR5-mediated immunosuppression in the active disease states of UC and CD.

### Depletion of bile acid-transforming pathways in the gut microbiome of patients with IBD

**Fig. 4:**
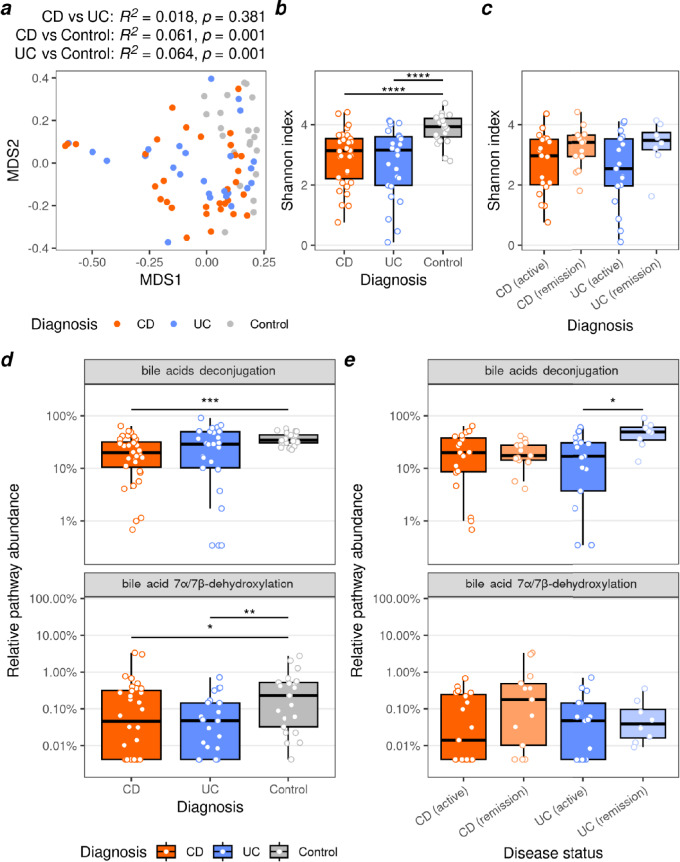
Gut microbiome diversity and abundances of bile acid-transforming pathways. (**a**) Multidimensional scaling (MDS) analysis of Bray-Curtis beta diversities and group separation analysis through Permutational Multivariate Analysis of Variance (PERMANOVA) using distance matrices. Pairwise PERMANOVA p-values and R^2^ values are stated on top. (**b**) Microbiome alpha diversities calculated as the Shannon index and stratified by diagnosis (Crohn’s Disease [CD], ulcerative colitis [UC], and healthy controls [Control]) and (**c**) stratified by disease status (active disease vs remission). (**d**) Relative abundances of bile acid-transforming pathways stratified by diagnosis and (**e**) stratified by disease status. Bars above box plots indicate significant differences between the two groups at each end of the bar (Wilcoxon rank-sum test with continuity correction). Significance levels: * (p < 0.05), ** (p < 0.01), *** (p < 0.001), **** (p < 0.0001).

Given that bile acid composition in both plasma and stool is shaped by host-microbiome co-metabolism, we used stool metagenomic sequencing to assess the relative abundance of microbial species and microbial bile acid transforming pathways in patients with IBD and healthy controls. We observed that the microbiome composition significantly differed in UC and CD when compared to healthy controls (Permutational Multivariate Analysis of Variance (PERMANOVA); CD vs controls: *R*^*2*^ = 0.061, *p* = 0.001; UC vs controls: *R*^*2*^ = 0.064, *p* = 0.001; Fig. 4a). Both CD and UC, displayed microbiome compositions with significantly lower alpha diversity (i.e., Shannon index) compared to the control group (Wilcoxon rank-sum test, *p*_*CD*_ = 4.2x10^−5^, *p*_*UC*_ = 8.9x10^−5^, Fig. 4b). No significant differences in alpha diversity were observed between CD and UC patients in remission and those with active disease, respectively. However, when considering all patients with IBD (UC and CD combined), patients with active disease had reduced microbiome alpha diversity compared to patients in remission (Wilcoxon rank-sum test, *p* = 0.0341).

CD samples showed a significantly reduced abundance of the microbial pathway responsible for bile acid deconjugation compared to healthy controls (Wilcoxon rank-sum test, *p*_*CD*_ = 0.0003), while both CD and UC samples exhibited reduced levels of the pathway mediating bile acid dehydroxylation (*p*_*CD*_ = 0.0449, *p*_*UC*_ = 0.0072, Fig. 4d). Among UC patients, those with active disease had significantly lower levels of the deconjugation pathway compared to those in remission (*p*_*UC*_ = 0.0182, Fig. 4e).

**Fig. 5:**
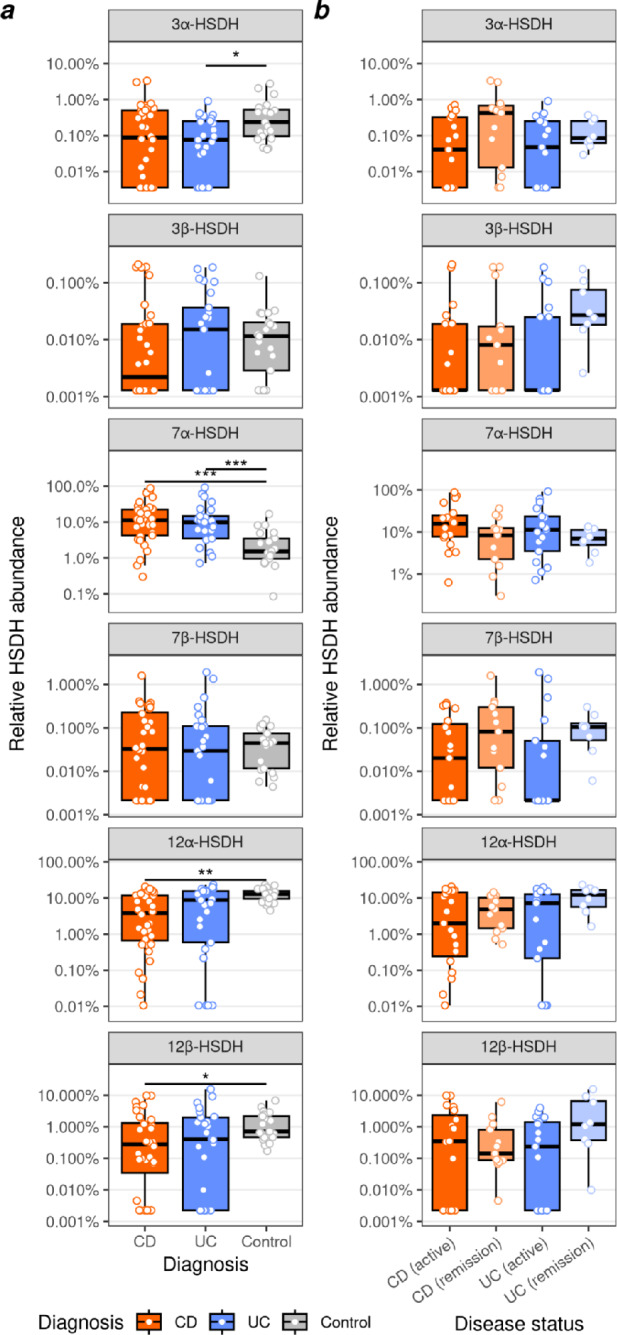
Relative abundances of gut microbial genes for hydroxysteroid dehydrogenase (HSDH) enzymes. (**a**) The data are stratified by diagnosis (Crohn’s Disease [CD], ulcerative colitis [UC], and healthy controls [Control]) and (**b**) by disease status (active disease vs remission). Bars above box plots indicate significant differences between the two groups at each end of the bar (Wilcoxon rank-sum test with continuity correction). Significance levels: * (p < 0.05), ** (p < 0.01), *** (p <0.001), **** (p < 0.0001).

At the enzyme level, the gene encoding 3α-hydroxysteroid dehydrogenase (3α-HSDH) was significantly less abundant in UC patients relative to controls (*p*_*UC*_ = 0.0062, Fig 5a), with a similar trend, although not significant, observed in CD patients (*p*_*CD*_ = 0.059). This reduction was even more pronounced in patients with active disease (Fig. 5b). In CD, the abundance of 12α-HSDH and 12β-HSDH was also significantly decreased compared to healthy controls (*p*_*CD*_ = 0.0246, Fig. 5a). Interestingly, 7α-HSDH displayed a distinct pattern, with significantly increased abundance in both UC and CD compared to controls (*p*_*CD*_ = 2.9x10^−5^, *p*_*UC*_ = 0.0001), particularly in active disease (Fig. 5a–b), suggesting a possible shift in microbial bile acid metabolism pathways in the context of intestinal inflammation.

To identify the microbial taxa underlying these pathway-level changes, we next examined the species contributing to bile acid transformation (Tables S3–S5). Bile acid deconjugation was predicted to be a widespread metabolic function, with 1,484 of 3,758 profiled microbial species harboring the corresponding pathway. Consequently, the reduced abundance of the deconjugation pathway in CD could not be attributed to the loss of a single dominant species but rather reflected a community-wide reduction in bile acid-deconjugating bacteria. Although the largest decreases were observed for *Agathobacter faecis* and *Agathobacter rectalis* (Table S3), these species accounted for only a small fraction of the overall reduction in the predicted abundance of the deconjugation pathway, which decreased from a median of 35% of the microbiome in controls to 20% in CD patients (Fig. 4d).

In contrast, only a small fraction of the gut microbiome was predicted to possess the 7α/7β-dehydroxylation pathway (median relative abundance: 0.23% in controls vs. 0.05% in both UC and CD; Fig. 4d). The reduction in IBD was primarily driven by depletion of a small number of uncultivated bai-carrying taxa, predominantly members of the Oscillospiraceae CAG-103 lineage (*CAG-103 sp000432375*, *CAG-103 sp900757655*, and *CAG-103 sp900317855*), together with *Lachnoclostridium_B sp900066555* (Table S4). In contrast, the cultivated bile acid 7α-dehydroxylating species *Clostridium_AP scindens* was not depleted and showed a higher prevalence in UC (28%) and CD (31%) than in healthy controls.

The abundance of the 7α-HSDH enzyme showed the opposite pattern, increasing from a median of 1.5% of the microbiome in controls to 11.2% in CD and 9.9% in UC (Fig. 5). Similar to bile acid deconjugation, this increase reflected the cumulative expansion of multiple 7α-HSDH-carrying taxa rather than a single dominant species, although the largest increase was observed for the *Escherichia coli_D* lineage (Table S5).

### Metagenome-predicted bile acid transformation pathways are associated with fecal and circulating bile acid profiles

To investigate whether the predicted microbial bile acid transformation capacity was reflected in the measured bile acid composition, we correlated the relative abundance of bile acid-transforming pathways with fecal and plasma bile acid concentrations.

The most consistent associations were observed for the bile acid 7α/7β-dehydroxylation pathway. Across sample types, higher pathway abundance was positively associated with dehydroxylated bile acids, whereas no positive associations were observed for primary or conjugated bile acids (Fig S3 and S4). In both UC and CD patients, the abundance of the dehydroxylation pathway correlated significantly with total dehydroxylated bile acids (stool: UC, p = 0.0002; CD, p = 0.0015; plasma: CD, p = 7.4x10⁻⁷). In CD patients, these associations were primarily driven by DCA (plasma: p = 7.4x10⁻⁷; stool: p = 0.02), GDCA (plasma: p = 1.6x10⁻^5^; stool: p = 0.006), and LCA (stool: p = 7.2x10⁻^5^). In UC patients, fecal DCA (p = 0.0006) and LCA (p = 0.0007) showed the strongest positive correlations with the abundance of the bile acid dehydroxylation pathway (Fig. S3–S4).

In contrast, the predicted abundance of bile acid deconjugation, 3α-HSDH, 12α-HSDH, and 12β-HSDH was predominantly associated with reduced concentrations of primary deconjugated and conjugated bile acids in stool samples from patients with CD. In CD patients, higher abundance of these pathways was significantly associated with lower fecal levels of the conjugated bile acids GCA, GCDCA, TCA, and TCDCA, as well as the deconjugated primary bile acids CA and CDCA (Fig. S4).

Together, these findings demonstrate that metagenome-predicted microbial bile acid transformation pathways are reflected in both fecal and circulating bile acid profiles, with the strongest and most specific associations observed for microbial 7α/7β-dehydroxylation.

## Discussion

Dysbiosis, defined as an imbalance in the intestinal microbiome, plays a crucial role in IBD pathophysiology^21,22^. Among microbiome-derived metabolites, secondary bile acids (sBAs) are of particular interest due to their regulatory effects on both innate and adaptive immunity^8,10,23^.

In our cohort of IBD patients, including those with active disease and in remission, we observed reduced microbial alpha diversity, accompanied by compartment-specific alterations in bile acid levels. Plasma and stool samples revealed a decreased sBA/pBA ratio, reflecting impaired microbial conversion of primary to secondary bile acids. Consequently, the bioactivity of TGR5, a G protein–coupled receptor counteracting intestinal inflammation, was diminished. Microbial pathway analysis confirmed a loss of bile acid–transforming capacity, particularly in enzymes involved in deconjugation and dehydroxylation.

Several studies consistently demonstrated dysregulated bile acid metabolism in active IBD, characterized by an increase in conjugated primary bile acids (e.g., TCA, GCA, GCDCA) and a decrease in secondary bile acids (e.g., LCA, DCA)^3,^^24-^^26^ Bile acids regulate epithelial integrity^27,28^, but accumulation of cholic acid (CA) can impair epithelial renewal and aggravate injury^29^. A simple categorization into “harmful” primary versus “protective” secondary bile acids is, however, misleading. While secondary bile acids (LCA, DCA) attenuate colitis^24,26,30,31^, some primary bile acids also exert protective effects^32,34,35^. Beyond epithelial effects, bile acids shape receptor-mediated mucosal immunity.

Although TGR5 has been implicated in IBD^9,10^, evidence on the impact of *in vivo*bile acid profiles was lacking. Prior studies examined individual bile acids^24,35^, but could not assess complex mixtures. Using a validated formula^19^, we estimated TGR5 activation by individual bile acid profiles. Fecal TGR5 activity inversely correlated with fecal calprotectin, indicating anti-inflammatory effects. Patients with reduced TGR5-activating bile acids in stool showed greater inflammation; similar findings were observed with circulating bile acids. Although total bile acid levels were slightly elevated in our patients, the lack of secondary bile acids was associated with reduced TGR5 activation, especially in active IBD. Our study is the first study to evaluate TGR5 bioactivity induced by complex bile acid profiles in IBD, underscoring its protective role in health and loss in disease.

TGR5 is highly expressed in myeloid cells, including monocytes, macrophages, and intestinal lamina propria cells^35,36^. Its activation suppresses proinflammatory cytokines, phagocytosis, and chemotaxis^37,38^. Our results align with findings from animal models: TGR5-deficient mice show aggravated colitis, while secondary bile acids — potent TGR5 agonists — ameliorate experimental colitis^24,26,31,39,40^. Likewise, fecal microbiota transplant from UC patients with reduced secondary bile acid capacity induced spontaneous colitis in antibiotic-pretreated mice^31^. By contrast, primary bile acids, which activate TGR5 only weakly^19,36^, attenuate colitis only at high doses^18,33^. In line with earlier findings^41,42^, our patients displayed increased primary bile acid levels, which, however, could not compensate for the lack of microbial secondary bile acid production. Beyond direct effects on innate immune cells, TGR5 influences downstream T-cell and dendritic cell responses and may also affect epithelial regeneration^43^. Nonetheless, myeloid cells remain central: bone marrow from TGR5−/− mice abolishes the protective effect of sBAs in wild-type recipients^24^.

Our findings emphasize the microbiota’s pivotal role not only in maintaining the pBA/sBA balance but also in shaping complex bile acid profiles relevant to inflammation. Supporting this concept, the abundance of metagenome-predicted bile acid transformation pathways correlated with the corresponding bile acid composition in both stool and plasma. In particular, the abundance of the 7α/7β-dehydroxylation pathway showed the strongest and most consistent positive associations with dehydroxylated bile acids, including DCA, GDCA, and LCA, indicating that the predicted microbial metabolic capacity is reflected in vivo by the measured bile acid pool (Fig. S3 and S4). Experimental studies have demonstrated that colonization of mice with *Clostridium scindens*, a cultivated 7α-dehydroxylating bacterium, alleviates colitis by increasing the production of the TGR5 agonists DCA and LCA^44^. Our findings support the importance of microbial 7α-dehydroxylation for maintaining the secondary bile acid pool but further suggest that, in humans, this metabolic capacity is primarily determined by uncultivated *bai*-carrying taxa. In our cohort, the reduced abundance of the 7α/7β-dehydroxylation pathway was mainly driven by depletion of members of the *CAG-103* lineage, whereas *C. scindens* itself was not depleted. In parallel, genes encoding 7α-hydroxysteroid dehydrogenase (7α-HSDH) were increased, potentially diverting CA and CDCA away from 7α/7β-dehydroxylation and thereby further limiting the formation of TGR5 agonists.

This study was designed as a proof-of-concept investigation to evaluate the plausibility of the recently proposed hypothesis of reduced TGR5 activity in patients with IBD. It is the first to translate the latest advances in TGR5 basic science research and animal studies into the clinical context, with the complex bile acid profiles found in humans. In this context, this study has several limitations. First and foremost, our hypothesis-driven study was focused on TGR5 activation and did not analyze the multifaceted effects of bile acids (e.g., FXR-mediated effects or direct effects on microbiota). Furthermore, we assessed TGR5 activity by a recently published formula which uses 15 bile acids commonly found in the circulation^19^. Accordingly, our results do not reflect the full spectrum of fecal and plasma bile acids found *in vivo*. Therefore, our study should be understood as a first step towards translating recent basic TGR5 research into the clinical context of IBD. Our finding, that patients with IBD show a loss of TGR5-activating bile acids, which is associated with disease activity, might pave the way for future studies targeting gut microbiota or directly modulating bile acids in these patients.

In summary, our study highlights the gut microbiota–bile acid–TGR5 axis as a key regulator of mucosal immune homeostasis. IBD-associated bile acid profiles, especially in active disease, are markedly less capable of activating TGR5 due to the decreased sBA/pBA ratio. Monitoring and modulating bile acid composition, TGR5 activity, and microbial conversion of primary bile acids to secondary bile acids may represent a promising individualized therapeutic strategy for IBD.

## Methods

### Study population

Patients undergoing outpatient and inpatient care at the Jena University Hospital’s Department of Internal Medicine with a confirmed diagnosis of Crohn’s disease (n=33) or ulcerative colitis (n=28) in either active disease flare or remission, were included in the study. Exclusion criteria were the use of bile acid sequestrants and any bowel-cleansing measures performed immediately prior to the sample collection as part of a colonoscopy. Disease activity was assessed using the Harvey Bradshaw Index (HBI) for known Crohn’s disease, and the Simple Clinical Colitis Activity Index (SCCAI) for known ulcerative colitis. Additionally, the Montreal classification of inflammatory bowel disease and the presence of extraintestinal manifestations were documented. Current IBD-specific medication, as well as other ongoing medications and any antibiotic use within the past 3 months, were recorded. Healthy control participants (n=21) were recruited under similar conditions. Exclusion criteria for the control group included the use of bile acid sequestrants, as well as a history of IBD. The characteristics of the patient and control populations are presented in Table 1.

### Sample collection

Stool samples were collected from patients and control participants using a commode specimen collector. Plasma samples were collected from a peripheral vein. Stool and plasma samples were stored at −80°C until analysis.

### Bile acid quantification

Fifteen individual bile acids in stool and plasma samples were measured (Table 2) by mass spectrometry using an LC-MS/MS in-house assay as described before^19^. Before measurement, stool specimens were diluted 1:3 in Dulbecco’s phosphate-buffered saline (Gibco, Thermo Fisher Scientific Inc., MA, USA) and homogenized for 30 s in a laboratory blender (Waring, Stamford, CT, USA). Subsequently, the stool suspension was centrifuged for 5 min at 16,000 g, and the supernatant was used for bile acid quantification. 30 µL of stool suspension or plasma was added to 85% aqueous methanol (Carl Roth, Karlsruhe, Germany) in a Thomson Single Step Filter vial (PES membrane 0.2 µM, Thomson Instrument Company, CA, USA) and mixed for 30 s. After centrifugation and filtration (200 g, 60 s), bile acids were determined using an Agilent 1200 high-performance liquid chromatography system (Agilent Technologies, Santa Clara, CA, USA) with an autosampler (PAL Systems, CTC Analytics AG, Zwingen, Switzerland) coupled to an API 4000 Triple Quadrupole mass spectrometer with electrospray ionization source (AB SCIEX, Framingham, MA, USA). All chromatographic separations were performed with a reverse-phase analytical column. The mobile phase consisted of water and methanol, both containing 0.012% formic acid (Merck KGaA, Darmstadt, Germany) and 5 mM ammonium acetate (Merck KGaA, Darmstadt, Germany), at a total flow rate of 300 µL/min.

### Estimate of TGR5 activation by bile acid profiles

TGR5 activity induced by each stool or plasma bile acid profile was calculated as described previously^19^. Briefly, the calculation assumes that the effects of individual bile acids on TGR5 activity are additive, with each bile acid contributing differently to the total activity. The following equation was used to estimate the TGR5 activity (*T*) in percent (%):

$$T={\sum}_{b\in B}\left({c}_{b}\times {P}_{b}\times k\right)$$

Where *b* denotes a specific bile acid from the set of all 15 considered bile acids *B* (Table 2), *c*_*b*_ is the concentration of bile acid *b* (in µmol/L), *P*_*b*_ is the potency of bile acid *b* to activate TGR5 (see Table 2), and k is a constant with the value 0.0085 (unit: %x L/µmol).

### Metagenome sequencing

Microbial DNA was extracted from fecal samples using the PureLink Microbiome DNA Purification Kit (Thermo Fisher Scientific, Waltham, MA) according to the manufacturer’s protocol. The quantity and quality of the isolated DNA from stool samples were assessed using the Qubit 2.0 and the Agilent TapeStation device. Library preparation was conducted using an Illumina Nextera XT DNA Library Preparation Kit (Illumina, San Diego, CA, USA) according to the manufacturer’s recommendations. The DNA underwent fragmentation and ligation with sequencing-specific adapters. End repair and A-tailing steps were performed to ensure proper adapter ligation. PCR amplification increased the material for sequencing, followed by purification and size selection using magnetic beads (AMPure XP, Beckman Coulter). For multiplexing, 10 bp indexes (IDT for Illumina DNA/RNA UD Indexes Set A, Tagmentation) were used to include all samples in a single library preparation. The resulting library was quality-controlled using an Agilent TapeStation device. Subsequently, the library was sequenced on an Illumina NovaSeq 6000 device using an Illumina NovaSeq 6000 S2 Reagent Kit v1.5 for 2 x 150 bp paired-end sequencing. Adapter removal and base trimming were performed using Illumina’s bcl2fastq tool, generating raw data in FASTQ format for downstream analysis (Table 3).

**Table 3:** Stool bile acids quantified in this study. Values for TGR5 activation potency are taken from Leonhardt et al.^19^. Values for bile acids are given in µmol/L.

	Bile acids	TGR5 potency (*P*_*b*_)	Crohn’s disease			Ulcerative colitis		
			Active disease	In remission	Overall	Active disease	In remission	Overall
Primary bile acids	CA	1.0						
	CDCA	8.4						
	GCA	1.2						
	GCDCA	7.0						
	TCA	1.4						
	TCDCA	8.1						
Secondary bile acids	DCA	28.0						
	GDCA	18.1						
	GLCA	180.6						
	GUDCA	1.2						
	LCA	28.0						
	TDCA	23.3						
	TLCA	243.5						
	TUDCA	1.8						
	UDCA	1.9						

CA, cholic acid; CDCA, chenodeoxycholic acid; DCA, deoxycholic acid; GCA, glycocholic acid; GCDCA, glycochenodeoxycholic acid; GDCA, glycodeoxycholic acid; GLCA, glycolithocholic acid; GUDCA, glycoursodeoxycholic acid; LCA, lithocholic acid; TCA, taurocholic acid; TDCA, taurodeoxycholic acid; TCDCA, taurochenodeoxycholic acid; TLCA, taurolithocholic acid; TUDCA, tauroursodeoxycholic acid; UDCA, ursodeoxycholic acid.

### Metagenome data processing

Metagenomic reads in FASTQ format were quality filtered using the QC workflow from the metagenome-atlas pipeline tool v2.18.0 with default parametrization^45^. The QC filtering included a step to remove reads from likely contamination sources, namely Illumina PhiX sequences and sequences originating from human DNA. To this end, the human reference genome assembly ‘Genome Reference Consortium Human Build 38’ (GRCh38) was used, in which low entropy regions (entropy < 0.7) were masked using the bbmask.sh tool within the BBmap suite. Moreover, regions that display high similarity to prokaryotic rRNA genes were additionally masked. To this end, prokaryotic small and large subunit rRNA gene sequences were retrieved from SILVA version 138.1^46^ and shredded into shorter (80 bp) sequences with 40 bp overlaps using shred.sh. Shredded sequences were aligned to GRCh38 with a minimum identity of 85% and a maximum indel length of 2 bp. Regions in GRCh38 with alignment hits were masked.

CoverM version 0.7.0^47^ was used to quantify the relative abundances of all 4824 HRGM2 representative genomes^48^. In brief, the tool aligns QC reads from the metagenomes to reference genomes (here: HRGM2). Relative abundances were estimated as the trimmed mean coverage (cover m option ‘-m trimmed_mean’) in the QC read alignment to the genomes sequences. This method calculated the average genome coverage by QC reads while the genome base pair positions with the highest 5% and lowest 5% coverages were removed before calculating the mean coverage.

Alpha diversity was calculated as the Shannon index^49^ based on the trimmed-mean coverage values for all HRGM2 genomes. Beta diversity was calculated using the Bray-Curtis dissimilarity index^50^ based on the relative abundances of HRGM2 genomes. Diversity metrics were calculated using the R-package vegan v2.7-1^51^. Multidimensional scaling (MDS) for two axes was applied to the calculated beta diversity values. Permutational multivariate analysis of Variance (PERMANOVA) was performed with 999 permutations based on the calculated beta diversities in pairwise comparisons (UC vs CD, CD vs Controls, and UC vs Controls) using the R-package pairwiseAdonis v0.4.1 commit cb190f7 (https://github.com/pmartinezarbizu/pairwiseAdonis).

### Prediction of gut microbial bile acid pathways and HSDH enzymes

To predict microbial pathways, the software gapseq (development version 1.5 commit 265f6cea) was used^52^. In detail, gapseq constructed genome-scale metabolic networks for all genomes of the HRGM2 reference genomes as described previously^53^ The reconstructed models were analyzed for the presence of two pathways relevant for the gut microbial transformation of primary to secondary bile acids, namely bile acids deconjugation (metacyc-ID: PWY-8135) and bile acid 7α/7β-dehydroxylation (PWY-7754). In addition, all models were tested for their presence of all six hydroxysteroid dehydrogenase (HSDH) enzymes that are involved in the epimerization of bile acids (Table S1). For each stool sample, the two bile acid pathways and the six enzymes were quantified by calculating the summed relative abundance of HRGM2 reference genomes that were predicted to encode the respective pathway/enzyme.

### Statistical data analysis

Statistical comparisons of variables among the patient groups (Crohn’s disease, ulcerative colitis, and controls) were performed using analysis of variance (ANOVA). For dichotomous variables (e.g., sex), comparisons were conducted using the chi-square test.

Bile acids that had more than 2/3 of the samples in all three diagnosis groups (Crohn’s disease, ulcerative colitis, and control) below the limit of detection were excluded from further analysis, except when calculating the TGR5 activation. For the remaining bile acids and for samples with concentrations below the limit of detection, a value of 0.05 µM was imputed.

Statistical data analysis involved pairwise group comparisons of microbial pathway and enzyme abundances, bile acid levels, and aggregated bile acid metrics (such as total secondary bile acids or TGR5 activation), which were conducted using Wilcoxon rank-sum tests in R v4.5.0^54^. Correlation between two continuous metrics were assessed using the Spearman method^55^. To account for multiple testing, p-values were adjusted using the method of Benjamini and Hochberg^56^. Unless otherwise specified in the presentation of results, p-values less than 0.05 were considered statistically significant. The graphical abstract was created with BioRender.com.

## Author contributions

A. Stallmach and J. Stallhofer designed and coordinated the study. J. Stallhofer, A. Steube, and J. Semmler obtained blood and stool samples and acquired clinical data; M. Ungelenk and C.A. Hübner performed metagenome sequencing; W. Löhden, L. Homeister, and S. Waschina processed metagenome data and analyzed gut microbial bile acid pathways; J. Semmler, S. Neugebauer, and M. Kiehntopf quantified bile acids; J. Leonhardt calculated TGR5 activity from bile acid profiles; J. Stallhofer, J. Leonhardt, S. Waschina, and A. Stallmach analyzed and interpreted the data and wrote the manuscript. All authors critically reviewed the final version of the manuscript and approved its submission.

## Funding

This work was supported by the Interdisciplinary Center for Clinical Research (IZKF) of the Jena University Hospital (Advanced Clinician Scientist Program ACSP 05 to J. Stallhofer and ACSP 12 to J. Leonhardt), the German Ministry of Education and Research (project code 01EJ2408C) to S. Waschina and by a grant (Hermann-Strauß-Forschungsstipendium) from the Deutsche Morbus Crohn/Colitis ulcerosa Vereinigung (DCCV e. V.) to J. Stallhofer.

## Supplementary Information

Supplementary Information 1.

Supplementary Information 2.

Supplementary Information 3.

Supplementary Information 4.

Supplementary Information 5.

## Data Availability

Metagenome sequencing data are provided via the European Nucleotide Archive ‘ENA’ (study accession: PRJEB101774). Bile acid quantification data and data analysis source code are available via ZENODO (https://doi.org/10.5281/zenodo.17465425).

## Abbreviations

BA: Bile acid
CD: Crohn’s disease
IBD: Inflammatory bowel disease
pBA: Primary bile acid
sBA: Secondary bile acid
UC: Ulcerative colitis
TGR5: Takeda G protein–coupled receptor 5
HSDH: Hydroxysteroid dehydrogenase
