# Supplementary figures and images for: Loss of TGR5-activating bile acids is associated with disease activity in inflammatory bowel disease
Source: Sci Rep. 2026 Jul 21;16:22812. doi: 10.1038/s41598-026-63156-0 (PMC13388663; doi:10.1038/s41598-026-63156-0)

Figure S1

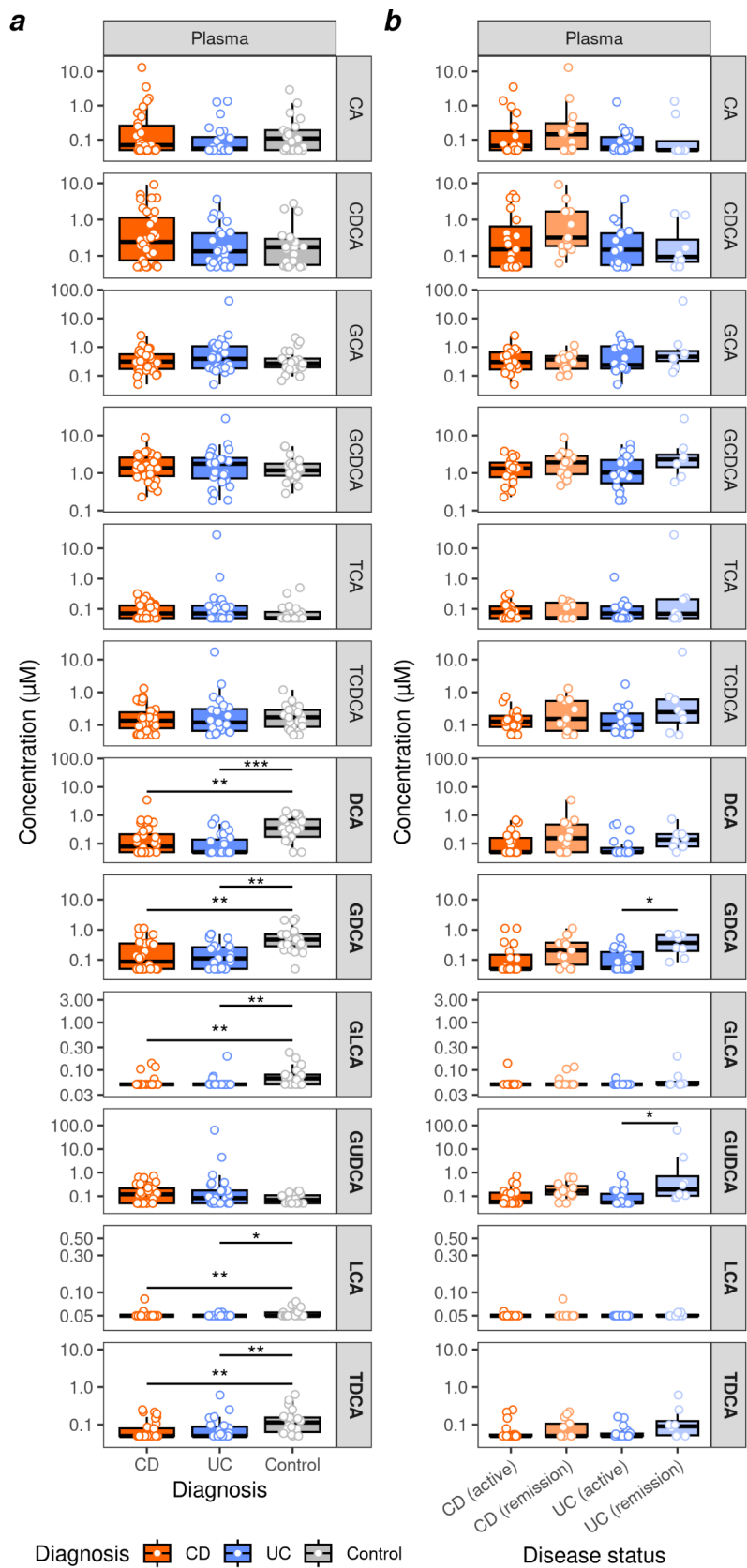

Supplement: Supplementary file 1 — Supplementary Information 1. [file 41598_2026_63156_MOESM1_ESM.pdf]

Figure S2

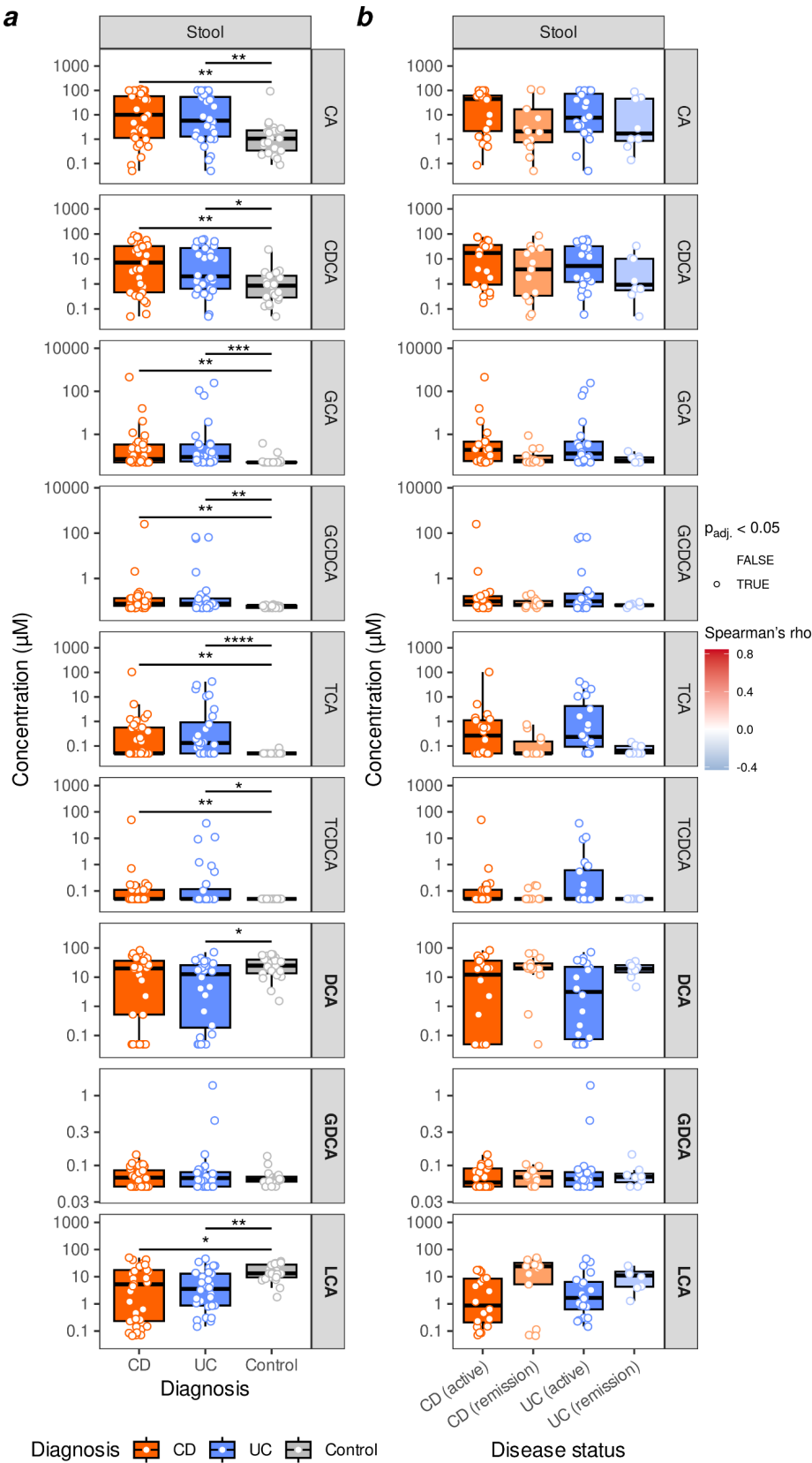

Supplement: Supplementary file 2 — Supplementary Information 2. [file 41598_2026_63156_MOESM2_ESM.pdf]

Figure S3

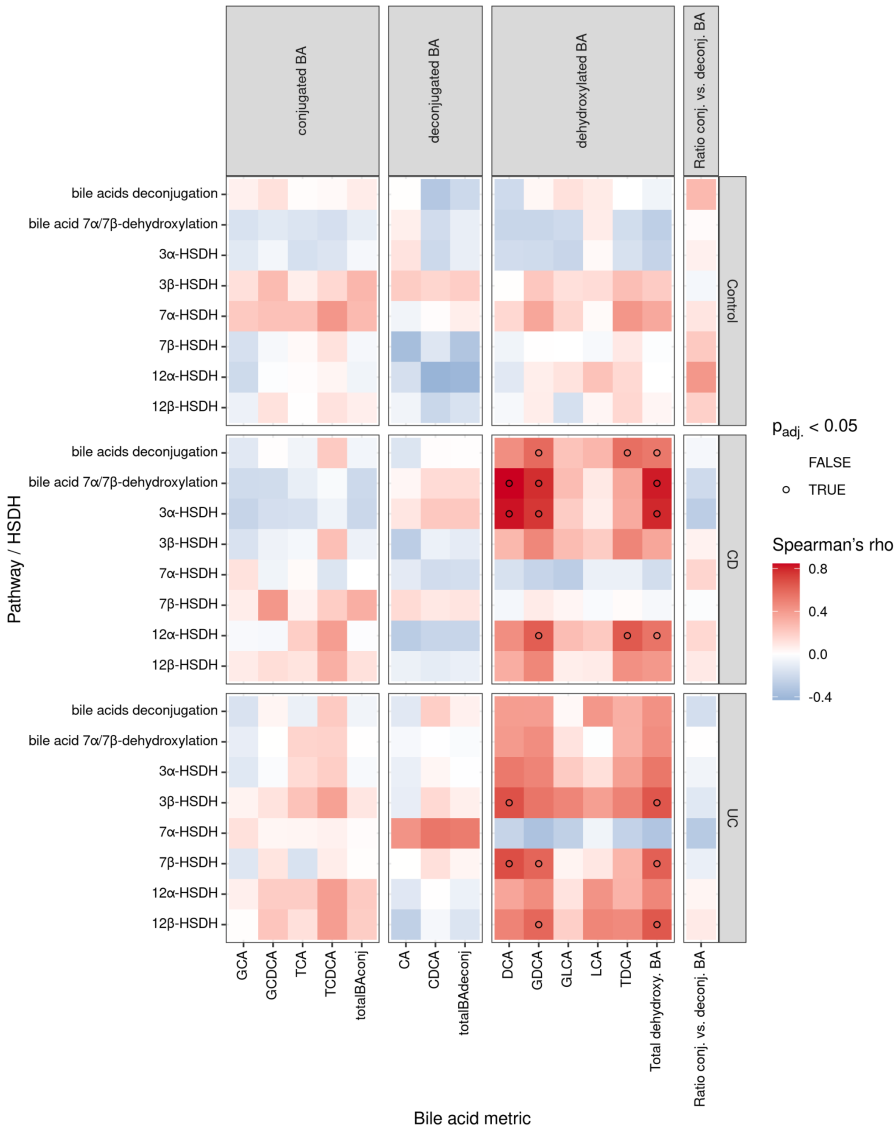

Supplement: Supplementary file 3 — Supplementary Information 3. [file 41598_2026_63156_MOESM3_ESM.pdf]

Figure S4

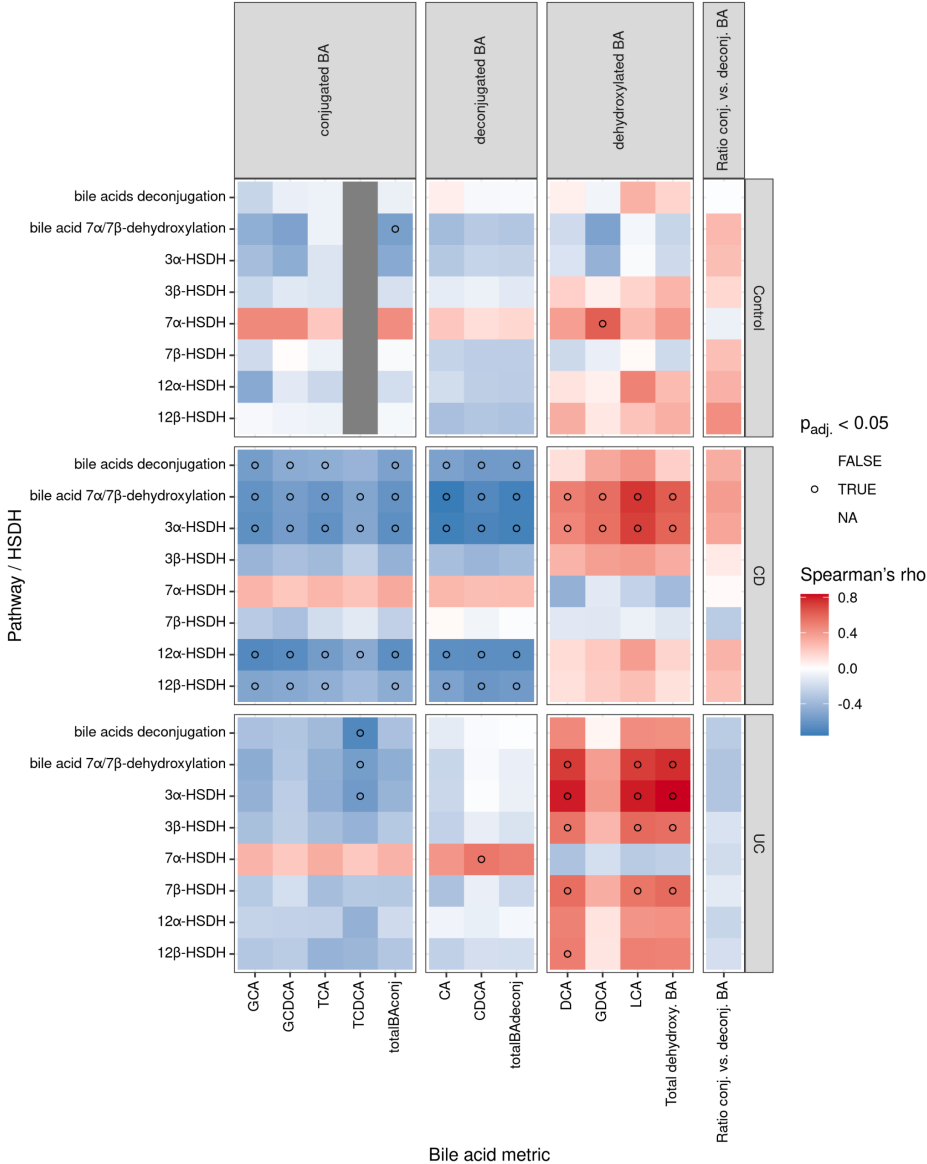

Supplement: Supplementary file 4 — Supplementary Information 4. [file 41598_2026_63156_MOESM4_ESM.pdf]
